# Editorial: Construction and biological applications of programmable DNA dynamic reactions

**DOI:** 10.3389/fchem.2023.1218742

**Published:** 2023-05-22

**Authors:** Shiquan Li, Ren Cai, Ding Ding, Edison Huixiang Ang, Yifan Lyu

**Affiliations:** ^1^ Molecular Science and Biomedicine Laboratory (MBL), State Key Laboratory of Chemo/Biosensing and Chemometrics, Aptamer Engineering Center of Hunan Province, College of Chemistry and Chemical Engineering, College of Material Science and Engineering, Hunan University, Changsha, Hunan, China; ^2^ State Key Laboratory of Oncogenes and Related Genes, Renji Hospital, Institute of Molecular Medicine (IMM), Shanghai Jiao Tong University School of Medicine, Shanghai Jiao Tong University, Shanghai, China; ^3^ Natural Sciences and Science Education, National Institute of Education Singapore, Nanyang Technological University, Singapore, Singapore

**Keywords:** DNA, dynamic reaction, self-assembly, aptamer, biosensing

Since the discovery of the molecular structure of DNA in 1953 by James Watson and Francis Crick, the programmability of DNA molecules and the predictability of DNA hybridization have garnered widespread attention. Benefiting from DNA solid-phase synthesis technology, desired DNA sequences can be conveniently synthesized to which functional molecules can be easily conjugated with declining costs. This drives the rapid development of DNA nanotechnology, including DNA dynamic reactions and DNA nanostructures. Additionally, the development of functional nucleic acids, such as aptamers (Bell et al., 2020) and DNAzymes (Ren et al., 2020), has allowed for the integration of DNA dynamic reactions in biological processes through molecular recognition-based conversion of biological signals into DNA signals. These advancements have led to the widespread use of DNA dynamic reactions in biosensing, bioregulation, and selective drug release in biological environments by responding to biological targets such as small molecules, nucleic acids, proteins, and cells (Gao et al., 2022; He et al., 2022; Tang et al., 2022; Yang et al., 2022). Moreover, the programmability of DNA also allows for the accurate assembly of customizable DNA nanostructures as functional and structural modules of DNA dynamic reaction, expanding the application potential of DNA nanotechnology. Based on the recent progress and the broad development prospects of DNA nanotechnology, this Research Topic focuses on the construction and biological applications of programmable DNA dynamic reactions and DNA nanostructures, especially in disease diagnostics, biomarker analysis, and drug delivery in complex bioenvironments. The bioanalytical and biomedical potential of DNA dynamic reactions and DNA nanostructures are emphasized in this Research Topic.

This Research Topic involves four papers, including two original research articles, one review, and one mini-review. The first paper of this Research Topic discussed the construction of transmembrane pores using DNA nanopores. Transmembrane transport is a crucial aspect of metabolic processes in living cells. Although the artificial construction of protein pores can provide powerful tools for biomedicine and bioregulation, the lack of general design rules for proteins has limited their development. DNA presents an excellent alternative compared to proteins as it is programmable and easy to synthesize. Several DNA nanopores have been reported thus far that can respond to various stimuli such as oligonucleotides, temperature, proteins, and light (Burns et al., 2016; Arnott and Howorka, 2019; Lanphere et al., 2021; Offenbartl-Stiegert et al., 2022). However, building DNA nanopores that can stay on the plasma membrane of living cells for extended periods remains a significant challenge. To address this Research Topic, Li et al. developed DNA biomimetic nanopores modified with ethane and phosphorothioate groups (PTT) that allowed for transmembrane transport on living cells. The single strand assembling the DNA nanopore was modified with hydrophobic phosphorothioate to anchor the DNA nanopore more securely to the plasma membrane of living cells, while positively charged ethane groups were modified to neutralize the electronegativity of the DNA nanopore. The study demonstrated that the DNA nanopores could remain on the plasma membrane of living cells for more than 1 hour at 37°C, longer than most DNA nanopores, and could transport Dox on tumor cells and drug-resistant tumor cells.

The second paper of this Research Topic discussed the use of aptamers to improve the targeting ability and treatment performance of nanomaterials. With the increasing use of nanomaterials in disease detection and treatment, improving their targeting ability remains a significant challenge. Many studies have focused on developing targeted delivery systems to enhance the efficacy of nanomaterials (Ouyang et al., 2020; Wan et al., 2022). One promising strategy is using aptamers, which are short, single-stranded oligonucleotides that can specifically bind to target molecules or cells with high affinity and selectivity. Cell-SELEX (systematic evolution of ligands by exponential enrichment) technology has been used to develop aptamers targeting a variety of cells (Zhang et al., 2012). These aptamers have the potential to improve the targeting ability of nanomaterials by facilitating their selective uptake into specific cells. Li et al. reported a platelet membrane-coated Prussian blue nanoparticle (PB) for synergistic photothermal therapy (PTT) and immunosuppression. The PB core has excellent photothermal conversion efficiency, while the platelet membrane coating efficiently protected the particles from immune clearance. The surface of the platelet membrane coating was modified with PD-L1 aptamer, AS1411 aptamer, and horseradish peroxidase (HRP) to achieve targeted and synergistic treatment. *In vitro* and *in vivo* experiments showed that the nanoparticle exhibited powerful antitumor effects on 4T1 cells under infrared irradiation.

The third article and fourth papers of this Research Topic reviewed the recent progress and applications of DNA dynamic reactions for biosensing, disease diagnosis, and biomimicry information processing. Zhang et al. reviewed the advancement of DNA-based functional modules that could be used for biomolecular signal sensing and transformation in biological systems. The design principles of different functional modules that can sense and convert target identification, concentration, order, duration, and spatial location into computable DNA signals were summarized and discussed. The obvious merits of DNA dynamic reactions in biological systems compared with silicon-based computing were emphasized and the challenges and limitations of DNA-based functional modules were evaluated. Mo et al. reviewed the recent progress and applications of two types of widely used isothermal and enzyme-free signal amplification strategies, hybridization chain reaction (HCR) and catalytic hairpin assembly (CHA). Biosensing strategies based on typical and advanced HCR and CHA strategies including branched HCR/CHA, localized HCR/CHA, and HCR/CHA-based cascaded reactions were discussed. They also summarized the limitations of HCR and CHA in biosensing and discussed potential solutions in the future.

## References

[B1] Arnott P. M. Howorka S. (2019). A temperature-gated nanovalve self-assembled from DNA to control molecular transport across membranes. Acs Nano 13, 3334–3340. 10.1021/acsnano.8b09200 30794375

[B2] Bell D. R. Weber J. K. Yin W. Huynh T. Duan W. Zhou R. (2020). *In silico* design and validation of high-affinity RNA aptamers targeting epithelial cellular adhesion molecule dimers. Proc. Natl. Acad. Sci. U. S. A. 117, 8486–8493. 10.1073/pnas.1913242117 32234785 PMC7165443

[B3] Burns J. R. Seifert A. Fertig N. Howorka S. (2016). A biomimetic DNA-based channel for the ligand-controlled transport of charged molecular cargo across a biological membrane. Nat. Nanotechnol. 11, 152–156. 10.1038/nnano.2015.279 26751170

[B4] Gao Y. Chen Y. Shang J. Yu S. He S. Cui R. (2022). Enzyme-free autocatalysis- driven feedback DNA circuits for amplified aptasensing of living cells. Acs Appl. Mater. Interfaces 14, 5080–5089. 10.1021/acsami.1c22767 35044153

[B5] He S. Yu S. Li R. Chen Y. Wang Q. He Y. (2022). On-site non-enzymatic orthogonal activation of a catalytic DNA circuit for self-reinforced *in vivo* MicroRNA imaging. Angew. Chem.-Int. Ed. 61, e202206529. 10.1002/anie.202206529 35775154

[B6] Lanphere C. Arnott P. M. Jones S. F. Korlova K. Howorka S. (2021). A biomimetic DNABased membrane gate for protein-controlled transport of cytotoxic drugs. Angew. Chem.Int. Ed. 60, 1903–1908. 10.1002/anie.202011583 PMC7894144 33231913

[B7] Offenbartl-Stiegert D. Rottensteiner A. Dorey A. Howorka S. (2022). A light-triggered synthetic nanopore for controlling molecular transport across biological membranes. Angew. Chem.-Int. Ed. 61, 8. 10.1002/anie.202210886 PMC10098474 36318092

[B8] Ouyang C. Zhang S. Xue C. Yu X. Xu H. Wang Z. (2020). PrecisionGuided missile-like DNA nanostructure containing warhead and guidance control for aptamer-based targeted drug delivery into cancer cells *in vitro* and *in vivo* . J. Am. Chem. Soc. 142, 1265–1277. 10.1021/jacs.9b09782 31895985

[B9] Ren W. Huang P.-J. J. de Rochambeau D. Moon W. J. Zhang J. Lyu M. (2020). Selection of a metal ligand modified DNAzyme for detecting Ni2+. Biosens. Bioelectron. 165, 112285. 10.1016/j.bios.2020.112285 32510338

[B10] Tang R. Fu Y.-H. Gong B. Fan Y.-Y. Wang H.-H. Huang Y. (2022). A chimeric conjugate of antibody and programmable DNA nanoassembly smartly activates T cells for precise cancer cell targeting. Angew. Chem.-Int. Ed. 61, e202205902. 10.1002/anie.202205902 35751134

[B11] Wan Q. Huang B. Li T. Xiao Y. He Y. Du W. (2022). Selective targeting of visceral adiposity by polycation nanomedicine. Nat. Nanotechnol. 17, 1311. 10.1038/s41565022-01249-3 36456644

[B12] Yang P. Guo X. Zhang J. Chen C. Gan Y. Xie W. (2022). Picomolar thrombin detection by orchestration of triple signal amplification strategy with hierarchically porous Ti3C2Tx MXene electrode material-catalytic hairpin assembly reaction-metallic nanoprobes. Biosens. Bioelectron. 208, 114228. 10.1016/j.bios.2022.114228 35367701

[B13] Zhang K. Sefah K. Tang L. Zhao Z. Zhu G. Ye M. (2012). A novel aptamer developed for breast cancer cell internalization. ChemMedChem 7, 79–84. 10.1002/cmdc.201100457 22170627 PMC3407573

